# Outcomes of a Near-Peer Intern Orientation Boot Camp

**DOI:** 10.7759/cureus.52126

**Published:** 2024-01-11

**Authors:** Rashid Alhusain, Astha Saini, Hersimren Minhas, Ahmed K Ahmed, Patrick Bishop, Baraa Osman, Hajra Khan, Omeralfaroug Adam, Jarrett J Weinberger, Diane L Levine

**Affiliations:** 1 Internal Medicine, Detroit Medical Center, Michigan, USA; 2 Internal Medicine, Wayne State University School of Medicine, Detroit, USA; 3 Internal Medicine, Wayne State University Detroit Medical Center, Detroit, USA; 4 Research, Mayo Clinic, Jacksonville, USA; 5 Internal Medicine, Detroit Medical Center/Wayne State University, Detroit, USA

**Keywords:** orientation, intern, near-peer, internal medicine, mentorship

## Abstract

Background: Interns experience challenges in their transition from medical school to residency. Orientation is traditionally delivered by faculty and administrators and often does not address practical skills needed by interns during the transition.

Objectives: The objective is to address traditional orientation gaps and improve incoming interns’ transition experience.

Methods: We identified opportunities with our intern orientation using a quality improvement methodology. Plan Do Study Act (PDSA) cycle 1 consisted of a pilot boot camp. PDSA cycle 2 was conducted over two weeks, June 9-23, 2021, at the Detroit Medical Center, Detroit, MI. Participation was voluntary. Residents were assigned incoming interns on a 1:1 basis. Five virtual sessions were conducted addressing: daily workflow, documentation, presentation skills, and utilization of the Electronic Health Record (EHR). All participants received pre- and post-program surveys.

Results: Twenty-two rising second- and third-year residents (26%) and 22 incoming interns (58%) participated. There was a significant improvement in the understanding of daily workflow (mean improvement 0.957, p=0.003), and most tasks associated with EHR including comfort with the sign-out process (mean improvement 1.21; p=0.002), accessing specific team lists (mean improvement 1.75, p=0.001), writing orders (mean improvement 1.41; p=0.002), composing documentation (mean improvement 1.23; p=0.001). Writing notes improved significantly (mean improved by 0.52; p=0.04). Nearly all (93.2%) stated the program achieved its overall goals and believed (92.9%) the program should be continued for incoming intern classes.

Conclusion: A targeted orientation bootcamp led by near-peers positively impacted the intern experience improving understanding of day-to-day responsibilities and comfort utilizing the electronic health record.

## Introduction

The transition from medical student to resident presents many challenges [1,2]. Senior electives and acting internships (sub-internships) provide opportunities for knowledge, skill acquisition, and clinical experience. Yet, the transition from medical school to residency can still be overwhelming. The Invitational Conference on USMLE Scoring (InCUS) co-sponsored by five national organizations (American Medical Association, Association of American Medical Colleges, Educational Commission for Foreign Medical Graduates, Federation of State Medical Boards, and National Board of Medical Examiners) identified transition challenges from undergraduate medical education (UME) to graduate medical education (GME) and the need for a national dialogue about educational transitions. This meeting underscored the need for a cross-organizational panel to address transition challenges from UME to GME [3]. As a result, the Coalition for Physicians Accountability (COPA) formed the UME to GME Review Committee (UGRC) which was charged with recommending solutions to challenges identified with this transition. The UGRC recommended “providing residents with a robust orientation at the start of internship which should include system utilization, introduction to the patient population, known health disparities, community service and engagement, faculty, peers, and institutional culture” [4].

The onboarding process addresses some of the COPA mandates, but primarily focuses on rules and regulations and duty responsibilities. The technical definition of “onboarding” is the process of integrating new employees into an organization. The Society for Human Resource Management (SHRM) divides the onboarding process into four levels--compliance, clarification, culture, and connection. Compliance and clarification are the two passive and lowest levels of the onboarding of the process and inform new members on policies and regulations and then ensure comprehension of the presented information [5]. Most residency programs have some form of departmental orientation that includes didactics to review medical knowledge and training on procedures paired with institutional onboarding [6-8]. Most residencies also have a formal process that provides incoming interns with documents or modules that delineate the rules and duties of residency and requires signatures confirming review and understanding of the material [9]. These informative documents/modules convey the culture of the residency program. However, they are unable to provide the last “C” that the SHRM considers the highest level of the onboarding process--connection. Connection refers to the interpersonal relationships that new members must form [5]. For new interns, the relationships formed with co-interns and senior residents are crucial for assimilation into a program as these near-peer relationships provide interns the safe space for open discussion [10]. Furthermore, there is literature that supports the benefit of near-peer education in residency [11-13].

Unfortunately, the COVID-19 pandemic presented barriers to making connections and created an additional layer of difficulty for incoming interns [14]. With the shift to virtual orientation sessions, one-on-one connections became more challenging to establish as digital platforms did not allow for mingling that would occur before or after in-person sessions.

To address both gaps in the traditional orientation and present-day challenges facing soon to be interns in internal medicine, we launched a voluntary near-peer bootcamp program in June 2020. We addressed gaps described in the UGRC and focused on system navigation and utilization including EHR education, daily duties, and workflow. We also addressed the "connection barrier" by utilizing near-peer relationships rather than faculty-intern relationships. We chose not to focus on community engagement or health equity as these topics are heavily integrated into the curriculum of our residency. Using a quality improvement process, we delivered the second iteration of the bootcamp in 2021. In this paper, we report the outcomes of our bootcamp.

This article was previously posted to Research square preprint server on July 6, 2022.

## Materials and methods

Pilot development and PDSA cycle 1: first orientation boot camp 

Our institution offers a formal orientation one week prior to the start of residency that includes a review of policies and procedures, an inpatient overview, communication skills, a tour of the facility, and training on the outpatient EHR. Formal evaluations of the orientation were positive but informal conversations suggested more was needed. 

In late 2019 we conducted a formal needs assessment through voluntary surveys, one-on-one interviews with current residents on topics and resources they would have liked to be covered during orientation. Additional input was provided from the Internal Medicine program leadership. We identified four key topics to address in the bootcamp: 1) daily workflow, 2) presentation skills, 3) documentation, and 4) utilization of the EHR. The bootcamp was organized and led by a senior resident and approved by the Internal Medicine Department leadership at Wayne State University. We piloted the near-peer orientation bootcamp in 2020. 

The interns in the class starting in July 2020 were offered participation in a voluntary near-peer boot camp entitled, “The Big Sibling Program”. Twenty-nine interns (76% of the program’s total interns) and fifteen senior residents signed up for the program. Senior residents were assigned to interns on a 1:2 basis (except for one resident who was paired with only one intern) and committed to delivering five sessions addressing the four topics areas described above. Sessions were conducted using a virtual format during the two weeks preceding the week-long formal orientation. The virtual platform was chosen to ensure social distancing and ease of access for incoming interns residing out of state. Both interns and seniors used their electronic devices for virtual communication. Sessions were arranged between residents and incoming interns at mutually convenient times. Most were conducted during evenings or weekends. All sessions were completed the weekend prior to beginning clinical duties and not during the week of the formal program orientation. Resources and educational materials were culled from online resources, previous in-house PowerPoint slide sets, and resident-developed resources. Resources were reviewed by resident leaders. After the pilot, a post-bootcamp survey was conducted to evaluate and assess intern perceptions, experience, and overall satisfaction with the boot camp.

PDSA cycle 2 and adaptation of session content 

In early 2021, we started planning for the 2021 boot camp. Based on a review of survey data from the 2020 boot camp and informal input from residents, we modified how interns were assigned to residents and modified the boot camp content. The signup form was modified to include questions related to personal background and career plans. The content was modified to include a discussion on how to study efficiently and how to identify and utilize available resources. We also further expanded content and skills related to the use of the EHR to introduce and review the three different inpatient and outpatient EHRs used by residents in our training program including using sign-out templates on the EHR and writing orders. Handouts were developed for each session to address objectives and were aligned with the Internal medicine milestones addressing the Accreditation Council for GME (ACGME) six core competencies: patient care, medical knowledge, systems-based practice, practice-based learning and improvement, professionalism, and interpersonal communication (Table 1) [15,16]. Seven additional resident leaders were recruited for the second PDSA cycle for a total of 22 leaders. Resident leaders were paired with interns on a 1:1 basis. Senior residents were assigned to incoming interns based on background and career plans.

**Table 1:** Cycle 2 Boot Camp sessions and objectives EHR: Electronic Health Record, PC-4: Patient management - inpatient, PC-5: Patient management - outpatient, PC-6: Digital Health, SBP-2: System Navigation for Patient-Centered Care, SBP-3 Physician Role in Health Care Systems, PBL-1: Evidence-Based and Informed Practice, PBL-2: Reflective Practice and Commitment to Personal Growth, Prof-3: Accountability/Conscientiousness

Session	Objectives	Internal Medicine Milestone
Daily Workflow	Provide an outline of a typical day on the wards. Discuss expectations, duties, and responsibilities of an intern. How to become efficient in performing your duties How to sign out your patients in an effective and safe manner Discuss expectations and providing feedback	SBP-2, Prof-3, PBL-2, ICS-2
Presentation Skills and Efficient Studying Techniques	Prepare interns to effectively present new patients Discuss different type of presentation; SOAP, problem-based, and system-based Discuss how to communicate with other services after placing a consult Identifying learning resources and protocols	PC-4, PC-5, SBP-2, SBP-3, PBL-1, Prof-3, ICS-2
EHR #1	Familiarize interns with different EHR systems they will be using. What to look for when reviewing charts Creating favorite order sets Creating macros Utilizing dictation system Accessing signout Who and how to reach out for EHR issues	PC-6, ICS-3
EHR #2	Familiarize interns with VA EHR (CPRS) How to optimize reviewing charts in CPRS How to compose a comprehensive note in CPRS Familiarize interns with ambulatory EMR	PC-6, ICS-3
Documentation	Discuss the importance of proper documentation Familiarize interns with different type of notes in the EHR Discuss the necessary components of HPI, SOAP, and discharge summary. How to write a note that has a proper clinical reasoning and appropriate for billing.	PC-6, SBP-2, Prof-3, ICS-3

Outcome evaluation methods 

Pre- and post-surveys (Appendix: S1) were collected through a secure online data collection tool (Qualtrics). Incoming interns were sent an informational email about the boot camp two weeks before the boot camp and asked to complete a pre-survey questionnaire to rate their level of comfort with certain tasks required by interns. Surveys were anonymous and no identifiable information was collected. Senior residents also received a pre-survey to assess their prior experience with mentorship. Items were rated using a 5-point Likert scale (1-very uncomfortable, 2-somewhat uncomfortable, 3-neither comfortable nor uncomfortable, 4-somewhat comfortable, and 5-very comfortable), and yes-or-no questions. A final free text box allowed participants to provide additional comments. After the conclusion of the bootcamp, incoming interns received a post-survey questionnaire to assess the knowledge and skills gained and satisfaction with the bootcamp. Senior residents received a post-survey to assess their experience with the boot camp.

Data from the pre- and post-surveys were entered and analyzed using IBM’s statistical package for social sciences (SPSS) version 28 (IBM Corp., Armonk, NY) [17]. Since only five mentees participated in both pre- and post-surveys, asymptotic significance (p-value) for independent non-parametric data was calculated using the Mann-Whitney U test. A cut-off point of 0.05 was used to determine statistically significant results. Eta squared $\eta ^2$ was used to estimate the effect size which is a quantitative measure of the strength of a phenomenon ($\eta ^2$ = 0.01 indicating a small effect; $\eta ^2$ = 0.06 indicating a medium effect; $\eta ^2$ = 0.14 indicating a large effect). The Wayne State University Institutional Review Board (IRB) reviewed the proposal and determined that it was an educational quality improvement, and that no IRB review was required.

## Results

A total of 22 senior residents and 22 interns (58% of the program’s total interns) participated. Of the interns who participated, 18 (82%) responded to the pre-survey (Appendix: S2) and 14 (64%) to the post-survey (Appendix: S3). Of the 14 interns, seven were mentored by post-graduate year (PGY)-2 residents and seven by PGY-3 residents.

Intern experience

**Figure 1:**
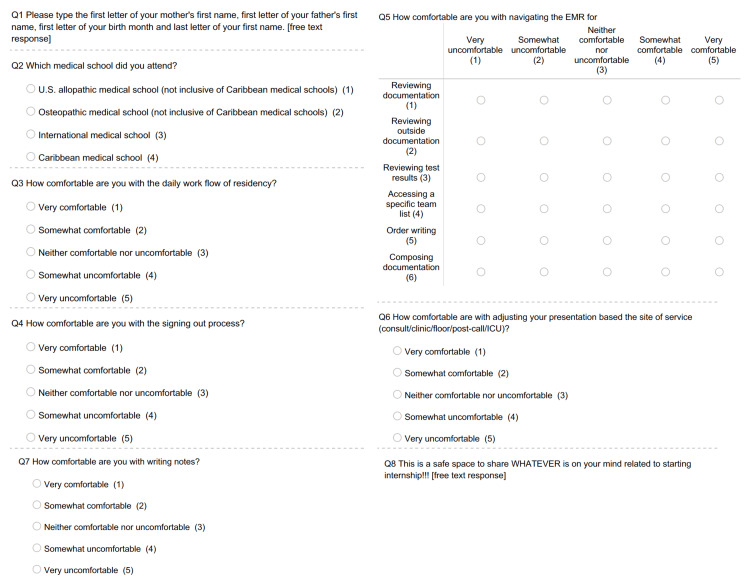
S1.1 - Incoming interns pre-survey

There was a statistically significant improvement in understanding of daily workflow (mean improvement 0.957, p-value=0.003, effect size ($\eta ^2$) = 0.288), and most tasks associated with EHR including comfort with the sign-out process (mean improvement 1.21; p-value = 0.002; effect size ($\eta ^2$) = 0.323), accessing specific team lists (mean improvement 1.75, p-value = 0.001, effect size ($\eta ^2$) =0.386), writing orders (mean improvement 1.41; p-value = 0.002; effect size = 0.315), composing documentation (mean improvement 1.23; p-value = 0.001; effect size = 0.330). Writing notes improved significantly however the effect size was to a lesser extent than other tasks (mean improvement 0.52; p-value = 0.04; effect size ($\eta ^2$) = 0.137). Interns’ comfort with presentation skills improved (mean improvement 0.37; p-value= 0.229; effect size ($\eta ^2$) = 0.047) but was not statistically significant (Table 2, Figure 1 in Appendix). Eleven interns (78.6%) felt more comfortable identifying study resources; nine interns (64.3%) stated that their mentor was effective in providing guidance; 12 interns (93.2%) stated that the program was effective in achieving its overall goals. Nine interns (64.3%) expressed that they are willing to participate as mentors for next year. Thirteen interns (92.9%) believed that the program should be continued for each incoming class of interns. Free text comments focused on themes of appreciation of one-on-one time with a senior resident, review of daily workflow, and the EHR (Appendix: S2 and S3).

Resident mentor experience

Of the 22 senior residents who participated, 11 (50%) responded to pre-survey (Appendix: S4) and seven (32%) responded to post-survey (Appendix: S5). Ten (90.9%) had previous experience with near-peer mentorship and 11 (100%) responded that they felt comfortable and prepared mentoring incoming interns. All senior residents stated that they felt comfortable participating in the program and providing mentorship. On a scale of very bad to very good, five (71.4%) rated their mentorship as very good. All respondents agreed that the program was effective in helping the interns with their new roles.

## Discussion

COVID-19 dramatically impacted the way medical education is delivered. Incoming interns were faced with less clinical experience and canceled elective rotations resulting in less preparation for internship [14]. As such, they had less time to acclimate to the workflow of a medical resident. COVID-19 also impacted the medical education of students in their Internal Medicine clerkships and sub-internships. It provided them with fewer opportunities to practice presentations, write notes, and place orders, all critical skills required to excel as interns. Our program helped to bridge this gap by giving incoming interns education addressing key skills that may not have developed or had been underdeveloped due to COVID-19. Our program also potentially addressed gaps present in the transition from UME to GME even prior to the onset of COVID-19. We demonstrated that reviewing the EHR helped interns with daily workflow. We also demonstrated a substantial improvement in interns’ comfort with tasks associated with EHR and to a lesser extent writing notes. Since we did not have a control group, we cannot compare those who participated and those who did not. Nevertheless, all respondents agreed that the program was helpful in preparing them for their duties as new interns.

The transition from medical school to residency is a challenging time. Accordingly, our institution and residency, and many others, provide a formal faculty-led orientation. The advantage of our near-peer orientation compared to the more traditional orientation stems from the social and cognitive congruence experienced between interns and senior residents, which according to Lockspeiser et al., allows for more productive and meaningful relationships [18]. Near-peer teaching compliments faculty teaching and may increase overall-reported preparedness [19,20]. Our sessions included a review of EHR use, including patient sign-outs, putting orders, composing notes, reviewing charts, outside medical records, and test results, all of which are integral components to the patient care competency and ultimate proficiency as an intern. Most importantly, our work followed COPA’s recent recommendations to provide residents with a specialty-specific orientation to increase preparedness and provide human connection with peers and near-peers.

Similar to the work by Beltman et al., our senior residents derived satisfaction from the program refining their teaching skills, developing stronger relationships with their coworkers, and fulfilling their role as mentors for the new interns [21]. Moreover, the program provided a platform for senior residents to practice leadership and communication skills. It has been reported that near-peer teaching serves as a medium for knowledge retention and improvement in public speaking and communication [10]. By allowing senior residents to volunteer their time towards the success and integration of new interns, we allowed senior residents to pay it forward as half of them were graduates of the prior year’s boot camp. Additionally, we individualized the experience for both residents and incoming interns by pairing them according to background and career plans. Furthermore, we exposed incoming interns to the benefits of near-peer teaching and found the majority were interested in participating as mentors.

Our work has several limitations. First, our near-peer boot camp was delivered to a single residency program and outcomes may relate to the interns recruited to our institution and residency. Second, participation in our program was voluntary. Incorporation into the required orientation might not have the same results as participation of self-selected participants. The virtual format, implemented because of COVID-19, also might not be as well received now that mandates for social distancing are no longer in place. Further studies are needed to determine the impact of the boot camp on the performance and efficiency of participants during their internship compared with those interns who did not participate to determine if this is to be recommended as a mandatory component of intern orientation.

## Conclusions

A targeted and structured orientation boot camp developed and led by near-peers positively impacted the intern experience by improving the level of understanding of day-to-day responsibilities, comfort utilizing the electronic health record, and promoting a sense of camaraderie and support. Our boot camp can serve as a model for other institutions, one that can be individualized based on specific residency needs. We encourage residencies to evaluate their orientations and consider addressing resident-identified gaps with a near-peer orientation boot camp.

## Figures and Tables

**Figure 2:**
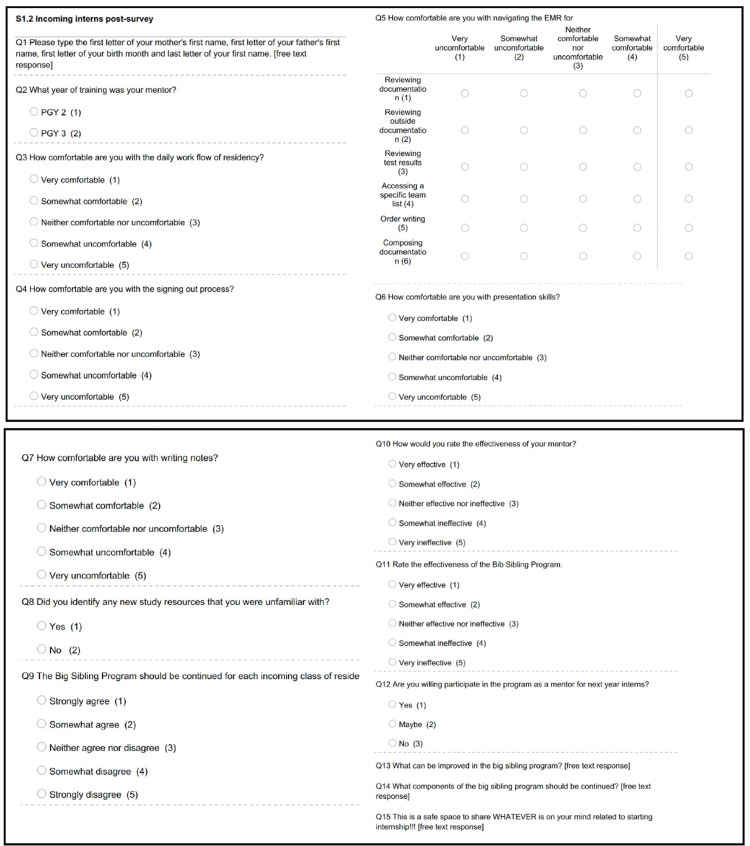
S1.2 - Incoming interns post-survey

**Figure 3:**
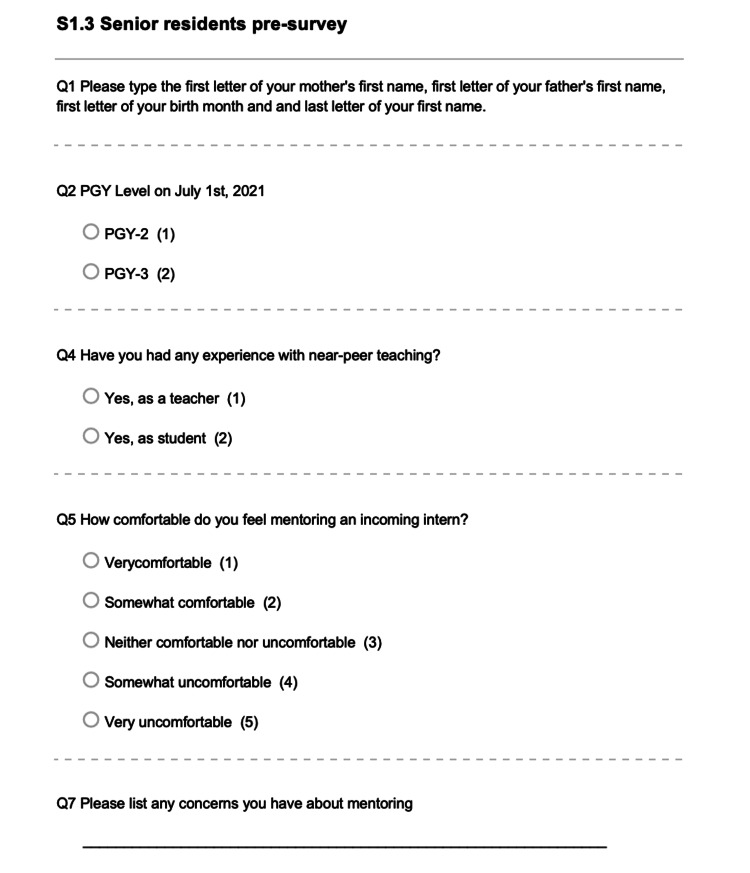
S1.3 - Senior residents pre-survey

**Figure 4:**
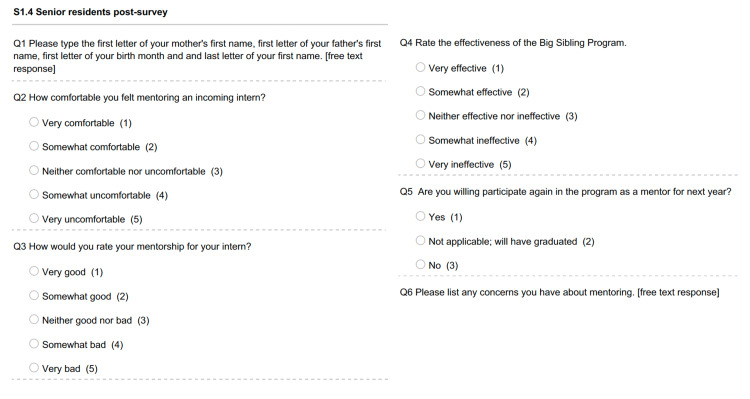
S1.4 - Senior residents post-survey

## Appendices

S1. Surveys

S2. Incoming interns pre intervention data

**Table 2:** S.2 - Incoming interns pre intervention data

Which medical school did you attend?	How comfortable are you with the daily work flow of residency?	How comfortable are you with the signing out process?	How comfortable are you with navigating the EMR for - Reviewing documentation	How comfortable are you with navigating the EMR for - Reviewing outside documentation	How comfortable are you with navigating the EMR for - Reviewing test results	How comfortable are you with navigating the EMR for - Accessing a specific team list	How comfortable are you with navigating the EMR for - Order writing	How comfortable are you with navigating the EMR for - Composing documentation	How comfortable are you with presentation skills?	How comfortable are you with writing notes?	This is a safe space to share WHATEVER is on your mind related to starting internship!!!
International medical school	Neither comfortable nor uncomfortable	Somewhat comfortable	Very comfortable	Somewhat comfortable	Very comfortable	Somewhat uncomfortable	Somewhat uncomfortable	Somewhat comfortable	Somewhat comfortable	Somewhat comfortable	I am familiar with the EMR as I used it in fellowship, but I anticipate that residency will be more demanding in different ways, so I hope to make the most of this initiative to better prepare me for the coming year. I'm both anxious and excited but looking forward to being a part of the IM family!
International medical school	Neither comfortable nor uncomfortable	Neither comfortable nor uncomfortable	Neither comfortable nor uncomfortable	Neither comfortable nor uncomfortable	Neither comfortable nor uncomfortable	Neither comfortable nor uncomfortable	Neither comfortable nor uncomfortable	Neither comfortable nor uncomfortable	Somewhat comfortable	Somewhat comfortable	
U.S. allopathic medical school (not inclusive of Caribbean medical schools)	Neither comfortable nor uncomfortable	Neither comfortable nor uncomfortable	Very comfortable	Somewhat comfortable	Very comfortable	Somewhat uncomfortable	Very uncomfortable	Somewhat comfortable	Very comfortable	Neither comfortable nor uncomfortable	I am excited and nervous to start. I hope that I can get some helpful advice on how to handle being an intern
Osteopathic medical school (not inclusive of Caribbean medical schools)	Neither comfortable nor uncomfortable	Somewhat uncomfortable	Neither comfortable nor uncomfortable	Somewhat uncomfortable	Neither comfortable nor uncomfortable	Somewhat uncomfortable	Somewhat comfortable	Somewhat uncomfortable	Somewhat uncomfortable	Neither comfortable nor uncomfortable	
Osteopathic medical school (not inclusive of Caribbean medical schools)	Somewhat uncomfortable	Somewhat uncomfortable	Very comfortable	Somewhat comfortable	Very comfortable	Neither comfortable nor uncomfortable	Neither comfortable nor uncomfortable	Somewhat comfortable	Neither comfortable nor uncomfortable	Somewhat comfortable	
Osteopathic medical school (not inclusive of Caribbean medical schools)	Somewhat uncomfortable	Somewhat uncomfortable	Somewhat comfortable	Somewhat uncomfortable	Somewhat comfortable	Somewhat uncomfortable	Very uncomfortable	Neither comfortable nor uncomfortable	Somewhat uncomfortable	Somewhat comfortable	
Osteopathic medical school (not inclusive of Caribbean medical schools)	Somewhat uncomfortable	Somewhat uncomfortable	Somewhat comfortable	Somewhat comfortable	Somewhat comfortable	Somewhat comfortable	Somewhat comfortable	Somewhat comfortable	Neither comfortable nor uncomfortable	Neither comfortable nor uncomfortable	
International medical school	Neither comfortable nor uncomfortable	Somewhat uncomfortable	Somewhat uncomfortable	Somewhat uncomfortable	Somewhat uncomfortable	Somewhat uncomfortable	Somewhat uncomfortable	Somewhat uncomfortable	Very comfortable	Neither comfortable nor uncomfortable	
Osteopathic medical school (not inclusive of Caribbean medical schools)	Neither comfortable nor uncomfortable	Somewhat comfortable	Somewhat comfortable	Somewhat uncomfortable	Somewhat comfortable	Somewhat uncomfortable	Very uncomfortable	Somewhat uncomfortable	Neither comfortable nor uncomfortable	Somewhat comfortable	
Osteopathic medical school (not inclusive of Caribbean medical schools)	Somewhat comfortable	Somewhat uncomfortable	Somewhat comfortable	Somewhat uncomfortable	Somewhat comfortable	Very uncomfortable	Very uncomfortable	Somewhat comfortable	Somewhat comfortable	Somewhat comfortable	
International medical school	Neither comfortable nor uncomfortable	Neither comfortable nor uncomfortable	Somewhat comfortable	Somewhat uncomfortable	Somewhat comfortable	Somewhat uncomfortable	Somewhat uncomfortable	Very uncomfortable	Somewhat comfortable	Somewhat comfortable	
International medical school	Somewhat uncomfortable	Somewhat uncomfortable	Neither comfortable nor uncomfortable	Somewhat uncomfortable	Neither comfortable nor uncomfortable	Very uncomfortable	Very uncomfortable	Somewhat uncomfortable	Somewhat uncomfortable	Somewhat uncomfortable	
International medical school	Neither comfortable nor uncomfortable	Neither comfortable nor uncomfortable	Somewhat comfortable	Somewhat uncomfortable	Somewhat comfortable	Somewhat uncomfortable	Neither comfortable nor uncomfortable	Neither comfortable nor uncomfortable	Somewhat comfortable	Neither comfortable nor uncomfortable	
U.S. allopathic medical school (not inclusive of Caribbean medical schools)	Somewhat uncomfortable	Somewhat uncomfortable	Neither comfortable nor uncomfortable	Somewhat uncomfortable	Neither comfortable nor uncomfortable	Somewhat uncomfortable	Very uncomfortable	Neither comfortable nor uncomfortable	Neither comfortable nor uncomfortable	Neither comfortable nor uncomfortable	
International medical school	Very comfortable	Very comfortable	Very comfortable	Very comfortable	Very comfortable	Very comfortable	Very comfortable	Very comfortable	Very comfortable	Very comfortable	
U.S. allopathic medical school (not inclusive of Caribbean medical schools)	Somewhat comfortable	Somewhat comfortable	Somewhat comfortable	Somewhat comfortable	Somewhat comfortable	Somewhat uncomfortable	Somewhat comfortable	Somewhat comfortable	Somewhat comfortable	Somewhat comfortable	
U.S. allopathic medical school (not inclusive of Caribbean medical schools)	Somewhat uncomfortable	Somewhat uncomfortable	Neither comfortable nor uncomfortable	Neither comfortable nor uncomfortable	Somewhat uncomfortable	Neither comfortable nor uncomfortable	Somewhat uncomfortable	Neither comfortable nor uncomfortable	Neither comfortable nor uncomfortable	Neither comfortable nor uncomfortable	
U.S. allopathic medical school (not inclusive of Caribbean medical schools)	Neither comfortable nor uncomfortable	Somewhat uncomfortable	Somewhat uncomfortable	Somewhat uncomfortable	Somewhat uncomfortable	Somewhat uncomfortable	Somewhat uncomfortable	Somewhat uncomfortable	Somewhat comfortable	Somewhat comfortable	Im excited!!

**Table 3:** S.3 - Incoming interns post-intervention data

What year of training was your mentor?	How comfortable are you with the daily work flow of residency?	How comfortable are you with the signing out process?	How comfortable are you with navigating the EMR for - Reviewing documentation	How comfortable are you with navigating the EMR for - Reviewing outside documentation	How comfortable are you with navigating the EMR for - Reviewing test results	How comfortable are you with navigating the EMR for - Accessing a specific team list	How comfortable are you with navigating the EMR for - Order writing	How comfortable are you with navigating the EMR for - Composing documentation	How comfortable are you with presentation skills?	How comfortable are you with writing notes?	How comfortable are you in identifying study resources?	The Big Sibling Program should be continued for each incoming class of residents.	How would you rate the effectiveness of your mentor?	Rate the effectiveness of the Bib Sibling Program.	Are you willing participate in the program as a mentor for next year interns?	What can be improved in the big sibling program?	What components of the big sibling program should be continued?	This is a safe space to share WHATEVER is on your mind related to starting internship!!!
PGY 3	Very comfortable	Very comfortable	Very comfortable	Very comfortable	Very comfortable	Very comfortable	Very comfortable	Very comfortable	Very comfortable	Very comfortable	Very comfortable	Strongly agree	Very effective	Very effective	Yes	I think that adding more sessions would help because it takes time to adjust to the EMR.	1) EMR training sessions 2) Presentation skills 3) How to writing a note 4) How to place orders	My mentor saved me. I was very nervous but I easily used Citrix on the first day, easily wrote my first note all because of her sessions with me.
PGY 3	Neither comfortable nor uncomfortable	Somewhat comfortable	Somewhat comfortable	Neither comfortable nor uncomfortable	Somewhat comfortable	Somewhat comfortable	Somewhat comfortable	Somewhat comfortable	Neither comfortable nor uncomfortable	Somewhat comfortable	Very uncomfortable	Strongly agree	Neither effective nor ineffective	Somewhat effective	Yes	My big sibling did not really reach out to me, I had to reach out to them and they didn’t really have any teaching sessions for me, I appreciate the concept and think it would be valuable, however perhaps more accountability from the mentors would be beneficial		
PGY 2	Neither comfortable nor uncomfortable	Neither comfortable nor uncomfortable	Neither comfortable nor uncomfortable	Somewhat uncomfortable	Somewhat comfortable	Very uncomfortable	Neither comfortable nor uncomfortable	Somewhat comfortable	Somewhat comfortable	Somewhat comfortable	Somewhat comfortable	Strongly agree	Neither effective nor ineffective	Very effective	Yes	Time spending prior to program start should be longer. Training topics should be structured and prep planed based on the program daily flow of work task and not based on what the incoming intern have to ask or have in doubts .		
PGY 2	Somewhat comfortable	Somewhat uncomfortable	Very comfortable	Very comfortable	Very comfortable	Very comfortable	Somewhat comfortable	Very comfortable	Somewhat comfortable	Somewhat comfortable	Somewhat comfortable	Strongly agree	Very effective	Very effective	Yes		Daily workflow discussion was really great and helped me feel more comfortable about starting	I have reached out to my big sib on multiple occasions since July 1. It has really helped make the transition a little more comfortable! I really appreciate this program!
PGY 3	Somewhat comfortable	Very comfortable	Very comfortable	Very uncomfortable	Very comfortable	Somewhat comfortable	Somewhat comfortable	Very comfortable	Somewhat comfortable	Very comfortable	Very comfortable	Strongly agree	Very effective	Very effective	Yes			
PGY 2	Somewhat comfortable	Somewhat comfortable	Very comfortable	Very comfortable	Very comfortable	Very comfortable	Very comfortable	Very comfortable	Somewhat comfortable	Very comfortable	Somewhat comfortable	Strongly agree	Very effective	Very effective	Yes			
PGY 2	Somewhat comfortable	Somewhat comfortable	Somewhat comfortable	Neither comfortable nor uncomfortable	Very comfortable	Very comfortable	Somewhat comfortable	Somewhat comfortable	Somewhat comfortable	Somewhat comfortable	Somewhat comfortable	Strongly agree	Very effective	Very effective	Yes			
PGY 3	Somewhat uncomfortable	Somewhat comfortable	Somewhat uncomfortable	Somewhat uncomfortable	Somewhat comfortable	Somewhat comfortable	Neither comfortable nor uncomfortable	Somewhat comfortable	Somewhat uncomfortable	Somewhat uncomfortable	Somewhat uncomfortable	Somewhat agree	Somewhat effective	Somewhat effective	Maybe			
PGY 3	Somewhat comfortable	Very comfortable	Somewhat comfortable	Somewhat comfortable	Somewhat comfortable	Very comfortable	Somewhat comfortable	Somewhat comfortable	Very uncomfortable	Somewhat uncomfortable	Somewhat comfortable	Strongly agree	Very effective	Very effective	Maybe			My mentor was amazing, super helpful. My assessments of my comfort levels above are mainly reflective of my adjustment process. She was fantastic as a mentor
PGY 2	Somewhat comfortable	Somewhat comfortable	Somewhat uncomfortable	Somewhat uncomfortable	Somewhat comfortable	Somewhat comfortable	Somewhat uncomfortable	Somewhat uncomfortable	Somewhat comfortable	Somewhat comfortable	Somewhat comfortable	Somewhat agree	Somewhat effective	Somewhat effective	Maybe			
PGY 3		Somewhat comfortable	Somewhat comfortable	Somewhat comfortable	Somewhat comfortable	Somewhat comfortable	Somewhat comfortable	Very comfortable	Somewhat comfortable	Somewhat comfortable	Very comfortable	Strongly agree	Very effective		Yes			
PGY 2	Somewhat comfortable	Somewhat comfortable	Somewhat comfortable	Somewhat uncomfortable	Very uncomfortable	Somewhat comfortable	Somewhat comfortable	Somewhat comfortable	Somewhat comfortable	Very comfortable	Neither comfortable nor uncomfortable	Somewhat agree	Neither effective nor ineffective	Somewhat effective	Maybe	Maybe expectations of what the big sibling can and should offer to the new intern.	It’s nice to have a one on one conversation with old intern before starting intern year	
PGY 2	Somewhat comfortable	Somewhat comfortable	Somewhat comfortable	Somewhat comfortable	Somewhat comfortable	Somewhat comfortable	Neither comfortable nor uncomfortable	Somewhat comfortable	Somewhat comfortable	Somewhat comfortable	Very comfortable	Neither agree nor disagree	Very ineffective	Somewhat ineffective	Maybe	More tangible resources and discussions, a lot of what was discussed did not really prepare me for the first month of intern year	Honestly not sure, I did not have the greatest mentor therefore cannot properly assess	
PGY 3	Very comfortable	Neither comfortable nor uncomfortable	Very comfortable	Somewhat comfortable	Very comfortable	Somewhat comfortable	Very comfortable	Very comfortable	Somewhat comfortable	Very comfortable	Somewhat comfortable	Somewhat agree	Somewhat ineffective	Very effective	Yes	organized review of information before starting residency, topics to review	questions, 1 on 1 guidance	

**Table 4:** S.4 - Senior residents pre-intervention data

PGY Level on July 1st, 2021	Have you been a mentor before?	Have you had any experience with near-peer mentorship?	How comfortable do you feel mentoring an incoming intern?	How would you rate your preparedness to mentor an incoming intern?	Please list any concerns you have about mentoring
PGY-2	No	Yes	Very comfortable	Very comfortable	
PGY-3	Yes	No	Very comfortable	Very comfortable	
PGY-2	Yes	Yes	Very comfortable	Somewhat comfortable	No concerns, this should be fun and a great way to get to know a colleague!
PGY-3	No	Yes	Somewhat comfortable	Very comfortable	
PGY-3	Yes	Yes	Very comfortable	Very comfortable	
PGY-2	Yes	Yes	Very comfortable	Very comfortable	
PGY-2	Yes	Yes	Very comfortable		
PGY-2	Yes	Yes	Very comfortable	Very comfortable	
PGY-3	Yes	Yes	Very comfortable	Very comfortable	
PGY-2	No	Yes	Somewhat comfortable	Somewhat comfortable	
PGY-3	Yes	Yes	Very comfortable	Very comfortable	

**Table 5:** S.5 - Senior residents post-intervention data

How comfortable you felt mentoring an incoming intern?	How would you rate your mentorship for your intern?	Rate the effectiveness of the Big Sibling Program.	Are you willing participate again in the program as a mentor for next year?	Please list any concerns you have about mentoring
Very comfortable	Very good	Very effective	Yes	
Very comfortable	Very good	Very effective	Yes	
Somewhat comfortable	Neither good nor bad	Somewhat effective	Yes	
Very comfortable	Very good	Very effective	Yes	No concerns. Great that there was a template for all mentors to follow and provided resources.
Very comfortable	Somewhat good	Somewhat effective	Yes	
Very comfortable	Very good	Very effective	Not applicable; will have graduated	
Very comfortable	Very good	Very effective	Yes	
